# Isolation and characterization of *Boudabousia marimammalium* from a mangrove habitat: identification of dual-function antimicrobial and anticancer peptides

**DOI:** 10.3389/fmicb.2025.1574859

**Published:** 2025-07-09

**Authors:** Yalpi Karthik, Srinivasa Krishnappa, Manjula Ishwara Kalyani, Ramakrishna D., Prabhudev S. H., Kumar K. M., Sanchit Thakur, Laila A. Al-Shuraym, Lamya Ahmed Alkeridis, Ohud Muslat Aharthy, Seham Sater Alhelaify, Muntazir Mushtaq

**Affiliations:** ^1^Department of Studies and Research in Microbiology, Mangalore University, Kodagu, India; ^2^Department of Studies and Research in Biochemistry, Mangalore University, Kodagu, India; ^3^Dr. C.D Sagar Centre for Life Sciences, Department of Biotechnology, Dayananda Sagar College of Engineering, Dayananda Sagar Institutions, Bengaluru, India; ^4^Department of Bioinformatics, Pondicherry University, Pondicherry, India; ^5^MS Swaminathan School of Agriculture, Shoolini University, Solan, India; ^6^Department of Biology, College of Science, Princess Nourah Bint Abdulrahman University, Riyadh, Saudi Arabia; ^7^Department of Biotechnology, Faculty of Science, Taif University, Taif, Saudi Arabia

**Keywords:** actinomycetes, antimicrobial peptides, anticancer peptides, *Boudabousia marimammalium*, mangrove habitat

## Abstract

Actinomycetes were isolated from mangrove soil samples and cultivated on starch casein nitrate (SCN) medium. The isolate was identified as *Boudabousia marimammalium* through 16S rRNA gene sequencing and phylogenetic analysis using MEGA X. Microscopic examination revealed filamentous mycelia with spirally coiled spore chains bearing cylindrical, hairy spores with curved edges, consistent with the genus morphology. Crude protein was extracted, partially purified (11.52-fold), and quantified (0.195 mg/mL, 8.48% recovery). Antimicrobial activity was tested against a panel of bacterial pathogens, and anticancer potential was evaluated using PC3 prostate cancer cells. LC-MS was employed for compound identification. The crude protein extract exhibited significant antimicrobial activity against *Proteus vulgaris* (22 ± 0.6 mm), *Salmonella typhimurium* (15 ± 0.6 mm), *Bacillus cereus* (13 ± 0.6 mm), *Pseudomonas aeruginosa* (14 ± 0.6 mm), and *Staphylococcus aureus* (10 ± 0.6 mm). It also showed anticancer activity, causing 37.43% growth inhibition of PC3 cells at 200 µg. LC-MS analysis identified a dipeptide with a molecular weight of 351.45 Da, corresponding to Tryprostatin B, a known bioactive compound. The isolate *B. marimammalium* from mangrove soil produces bioactive peptides with dual antimicrobial and anticancer properties.

## Introduction

Microbes are cosmopolitan in distribution throughout the world. Studies over a few decades reveal that the bioactive potential of microbes is known from ancient times (Berdy, 2005; Chater, 2013). Curiosity, genetic manipulation, and threats have increased our understanding of the significance and mechanisms of microbes (Buedenbender et al., 2017). Many biological impacts have proven that microbes alone, perhaps because of their ability to sustain extreme conditions, are a unique ability that has grabbed the attention of the scientific community for a sustainable future. Recently, researchers have focused on Actinomyces diversification studies, irrespective of habitat and biological system.

One such extreme habitat is the mangrove, which has an aberrant tidal frequency, salt content, limited nutrients, and unstable high-temperature conditions (Sivalingam et al., 2019; Sayed et al., 2020; Karthik and Kalyani, 2022). There are very limited studies that have reported on the mangrove region of Mangalore, as it remains an untapped area for microbial research (Aly et al., 2020). The diversity of such extreme habitat microbes shows potential metabolites for the treatment of various human ailments (Berdy 2005; Karthik et al., 2020). Few studies on novel and rare species in such extreme habitats have proven their potential for biological activities (Hoyles et al., 2001; Chen et al., 2015).

Actinomycetes are well known for ancient and modern therapeutics; they are involved in antibiotic production for antibacterial, antifungal, antiangiogenic, anticancer, antiparasitic, immunosuppressant, antihelminthic, enzyme inhibitor, immunomodulator, plant growth regulator, and wound healing activities (Janiszewska et al., 2012; Kalyani and Rajina, 2017; Herbrík et al., 2020; Karthik and Kalyani, 2021). A report states that about 45% of all bioactive secondary metabolites of microbial origin are obtained from the actinomycetes, and approximately 75% are produced by *Streptomyces* sp. (Abdillah et al., 2015; Karthik et al., 2023a, 2023b).

In addition, actinomycetes have the potential to produce various antibiotics. The recent problem of multidrug resistance and COVID-19 has led the world to identify new therapeutic agents from different reliable habitats (Kumar et al., 2023). This study identified one such rare and biologically evidenced important strain from the mangrove region of Mangalore: *Boudabousia marimammalium.*

## Materials and methods

### Soil sample collection sites

Soil samples were randomly collected from a mangrove habitat in the Mangalore region of Dakshina Kannada, Karnataka, India. The specific sampling site, Kallapu (KLPU), is located at 12°50′07.3”N, 74°51′31.2″E. Bacterial isolation was performed using the spread plate technique, and media optimization was carried out using distinct growth media, including Malt Extract Agar (MEA), Starch Peptone Agar (SPA), Glycerol Asparagine Agar (GAA), Nutrient Agar (NA), and Yeast Extract Agar (YEA). The 16S rRNA gene was amplified and sequenced, yielding a 986 bp fragment encompassing variable regions V3 to V9, which are widely recognized for their taxonomic informativeness. The sequence was analyzed using NCBI basic local alignment search tool, and phylogenetic analysis was conducted using MEGA X to infer evolutionary relationships. The procedures followed for bacterial isolation, media optimization, and molecular identification were based on the methodology described in our previous study (Karthik and Kalyani, 2022).

### Extraction of bioactive peptides from an actinomycete isolate

Actinomycetes strains were incubated at 30 ± 2°C for 7 days in starch casein agar (SCA) medium supplemented with 1% soya peptone under constant agitation at 100 rpm to promote growth. After incubation, the cultured biomass was harvested by centrifugation at 7000 rpm for 10 min. The cell pellet was washed twice with phosphate-buffered saline (PBS, Mg^2+^/Ca^2+^-free) to remove residual medium components and recentrifuged under the same conditions. The washed cells were then suspended in 10 mL of ice-cold acetone, thoroughly mixed, and incubated on ice for 5 min to precipitate proteins and disrupt cell membranes. Following this, the suspension was then centrifuged at 7000 rpm for 10 min, and the acetone supernatant was discarded. Residual acetone was removed by drying the pellet under a gentle stream of nitrogen gas to prevent protein denaturation. Finally, the protein fraction was solubilized by resuspending the dried pellet in 1.0 mL of 1% sodium dodecyl sulfate (SDS) and incubating for 2 min at room temperature, following the method of Bhaduri and Demchick (1983). The extracted protein was then stored at −20°C until further use.

### Antimicrobial activity

Antimicrobial activity was assessed using the agar well diffusion method. Wells (6 mm) were loaded with 50 μg of crude actinomycetes protein on nutrient agar plates inoculated with test pathogens (*E. coli, P. aeruginosa, K. pneumoniae, S. aureus, B. cereus, P. vulgaris, S. typhimurium,* and *E. aeruginosa*). Zones of inhibition were measured after 24 h of incubation.

### Protein purification and characterization

Actinomycetes protein was purified using Sephadex G-10 size-exclusion chromatography, equilibrated with 0.05 M sodium phosphate buffer (pH 7.0). Proteins were eluted at 10 mL/h, collected in 2-ml fractions, and tested for antibacterial activity via the well diffusion method. We analyzed the peak fraction from Sephadex G-10 using LC–MS (SynaptG2) with a Bridged Ethylene Hybrid C18 column (50 mm × 1.0 mm, 1.7 μm). Mobile phases were 0.1% formic acid in water (A) and 0.1% formic acid in acetonitrile (B), run on an Agilent 1,100 LC system. The anticancer assay (MTT) was performed as described by Karthik et al. (2023a).

## Results

**Figure 1:**
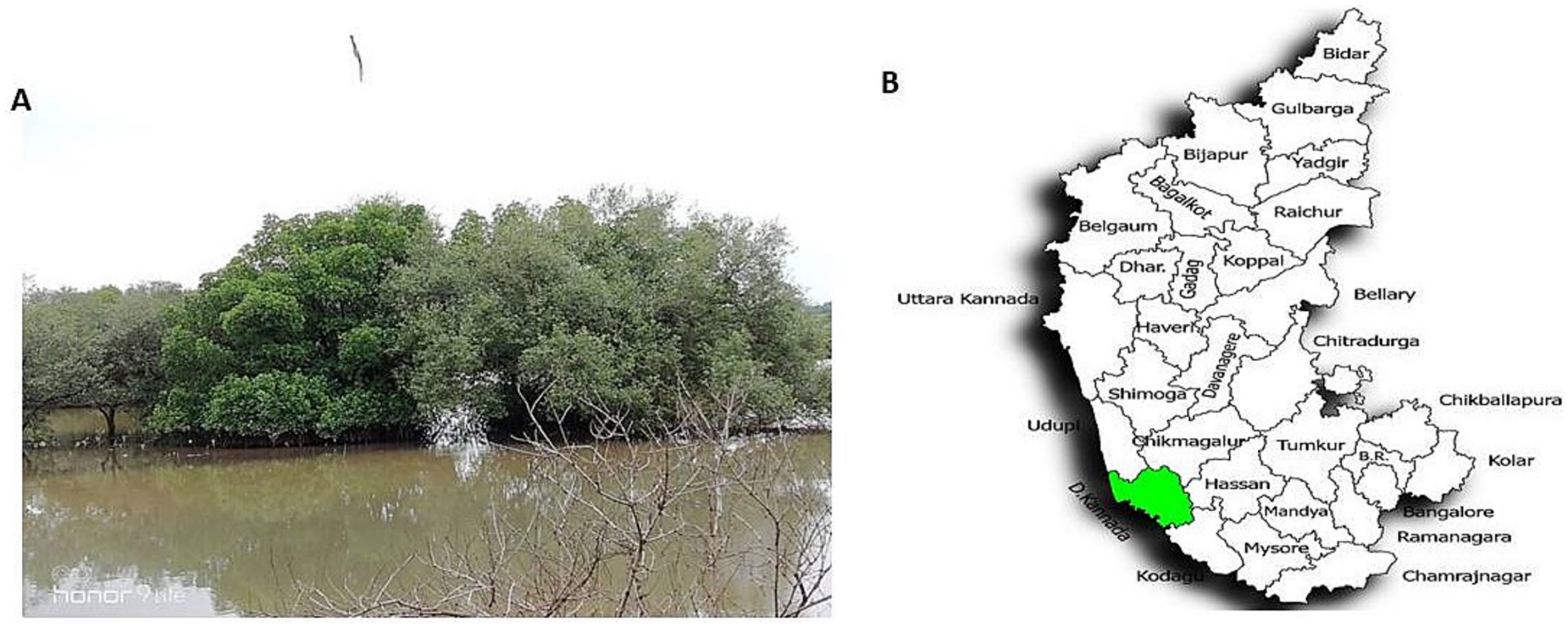
Mangrove soil sample collection site, Kallapu, Mangalore region. **(A)** Mangrove soil sample collection site, Kallapu, Mangalore region. **(B)** Location map depicting the soil sample collection in the Mangalore region of Dakshina Kannada, Karnataka, India.

**Figure 2:**
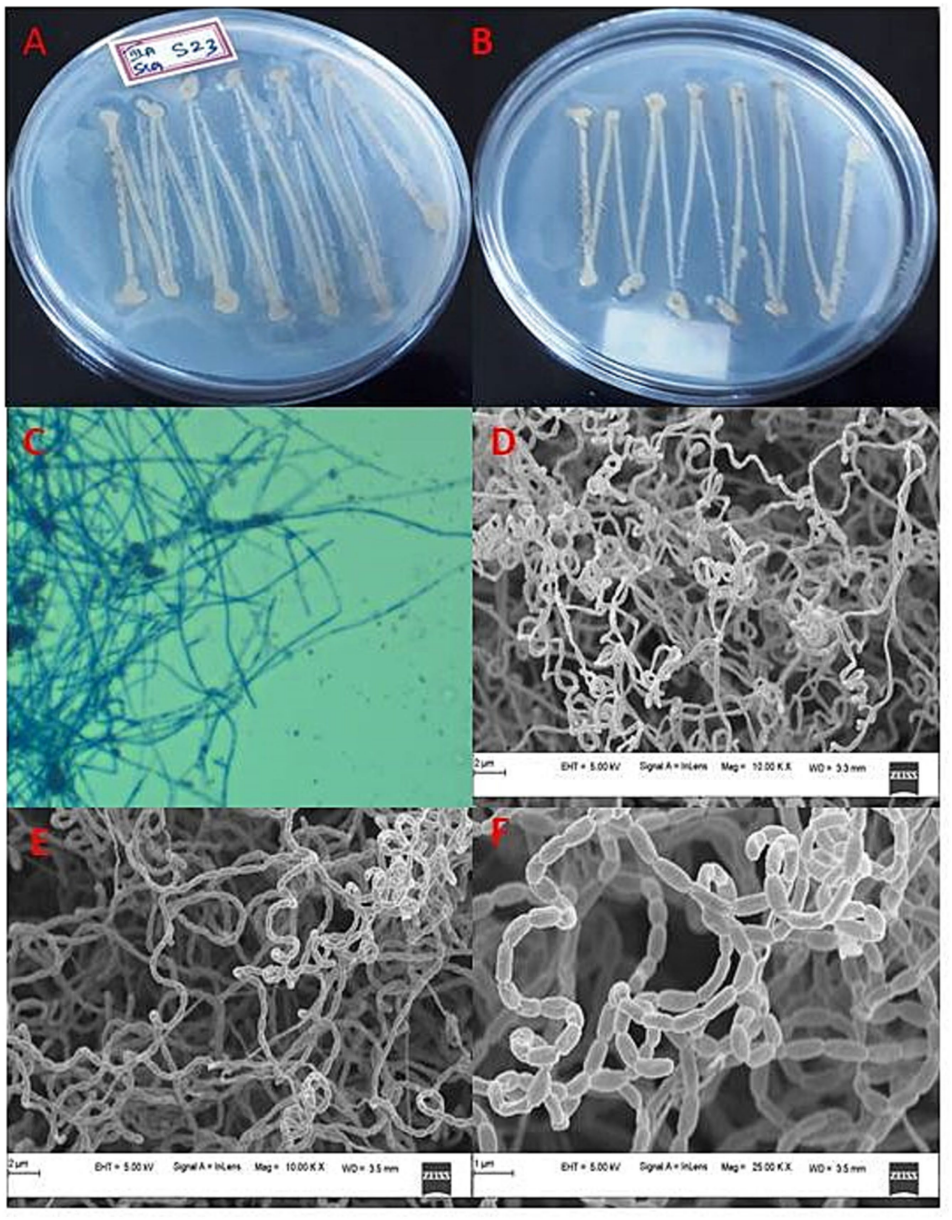
Cultural characteristics of actinomycete isolate S23: **(a)** Rear view of isolate, **(b)** Front view of isolate, **(c)** Phase contrast microscopic analysis, **(d)** Mycelia observations under FESEM, **(e)** Mycelia along with spore analysis under FESEM, and **(f)** Spore structure analysis using FESEM.

Mangrove habitats in Mangalore, specifically in Kallapu (Figure 1), provide a unique environment for isolating actinomycetes. The brown, powdery soil (21.2°C, pH 7.2) yielded isolate S23, which exhibited creamish-white mycelia and gray spores on maturity. S23 showed positive pigmentation on SCA media, with Gram-positive, spirally coiled spore chains and cylindrical, hairy spores (Figure 2). Growth optimization across the six media revealed excellent growth and gray spore production on starch casein nitrate, whereas other media showed limited or no growth (Table 1).

**Table 1:** Phenotypic characteristics of actinomycete isolates grown on different media (media optimization).

Isolate	Agar media	Growth	Front view	Rear view	Pigment	Spores
S23	Sucrose peptone	No	−	−	−	No
S23	Glucose leucine	No	−	−	−	No
S23	Nutrient agar	No	−	−	−	−
S23	Malt extract	No	−	−	−	No
S23	Yeast extract	Good	Cream	Creamish white	−	No
S23	Starch casein nitrate	Excellent	Brown	Black	+	Gray

### Molecular sequencing of actinomycete isolates

**Figure 3:**
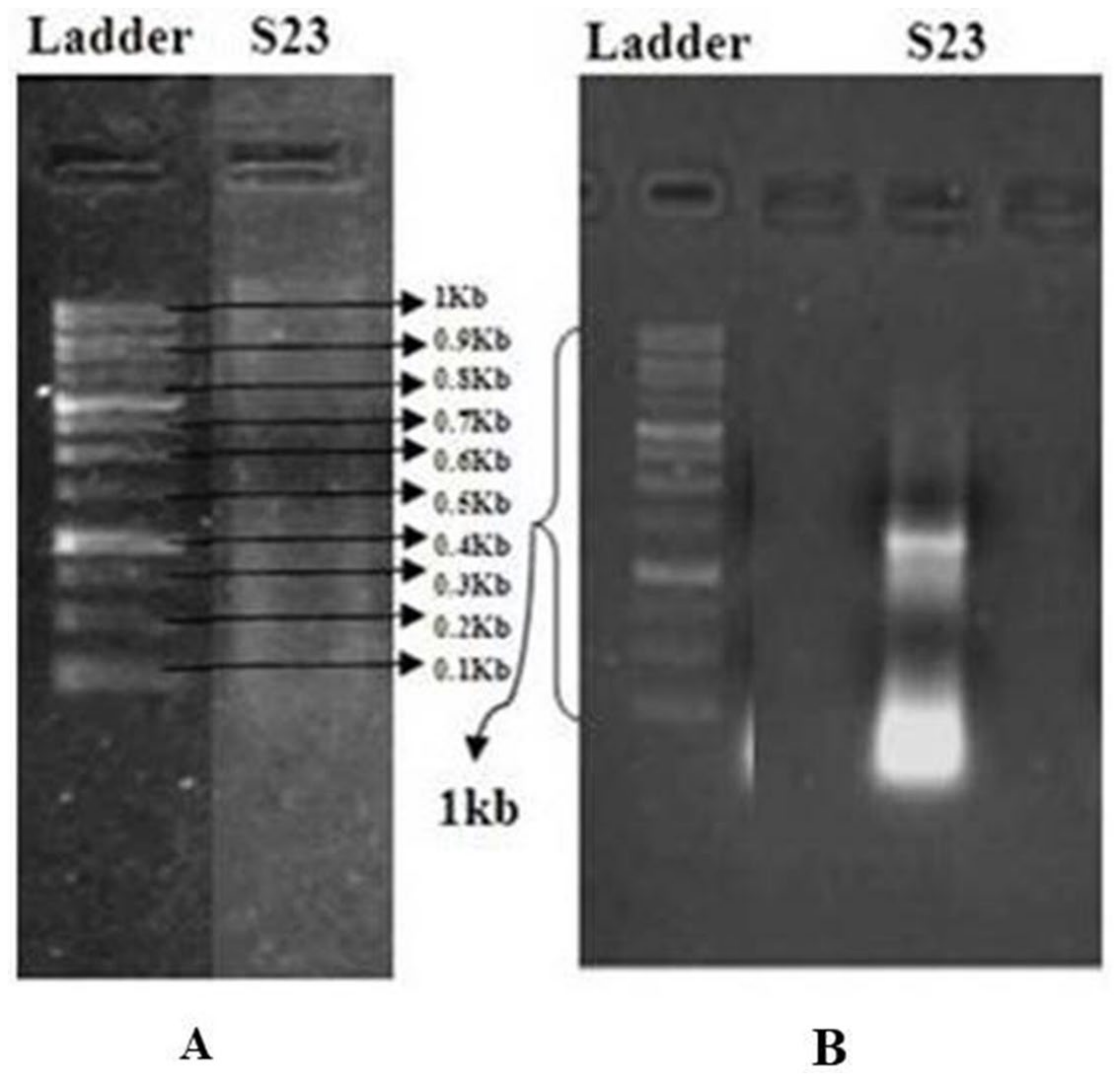
**(A)** Agarose gel electrophoresis for the detection of isolated DNA using the GeneRuler 1 kb DNA Ladder (Thermo Scientific) as a molecular weight marker. **(B)** Quantification of PCR-amplified DNA from a pure actinomycete isolate.

**Figure 4:**
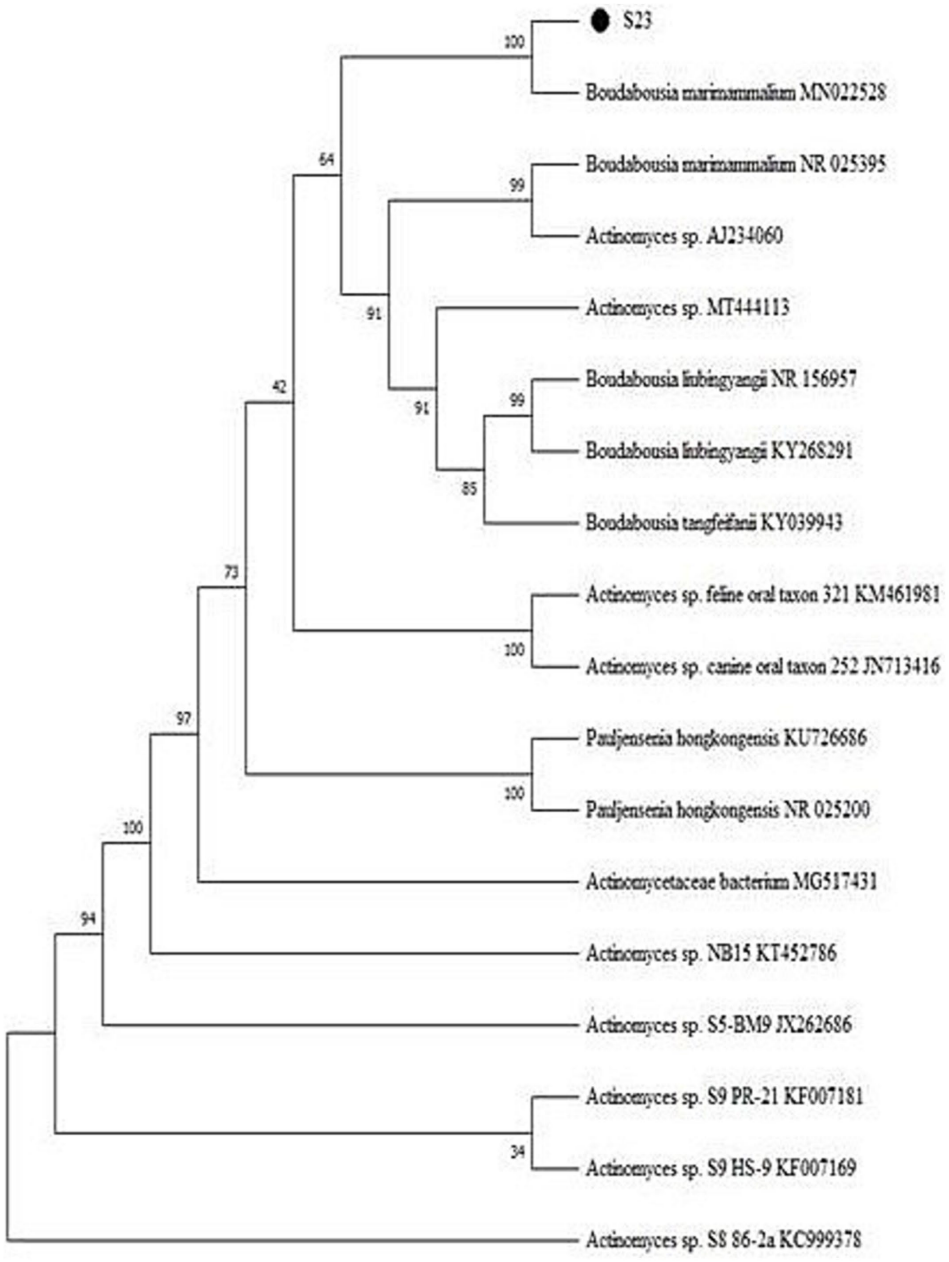
Phylogenetic tree of *Boudabousia marimammalium* YKIKM. MU03 (S23) using the Neighbor-Joining method.

Actinomycetes isolate S23 and its purified and amplified DNA (Figure 3) were identified as *Boudabousia marimammalium* YKIKM. MU03 (GenBank: MW898115). The phylogenetic tree (Figure 4) and maximum-likelihood species confirmed this identification. Previously reported in marine mammals in the UK (Hoyles et al., 2001), this study marks the first report of *B. marimammalium* from the mangroves of Mangalore.

### Protein extraction and purification

**Figure 5:**
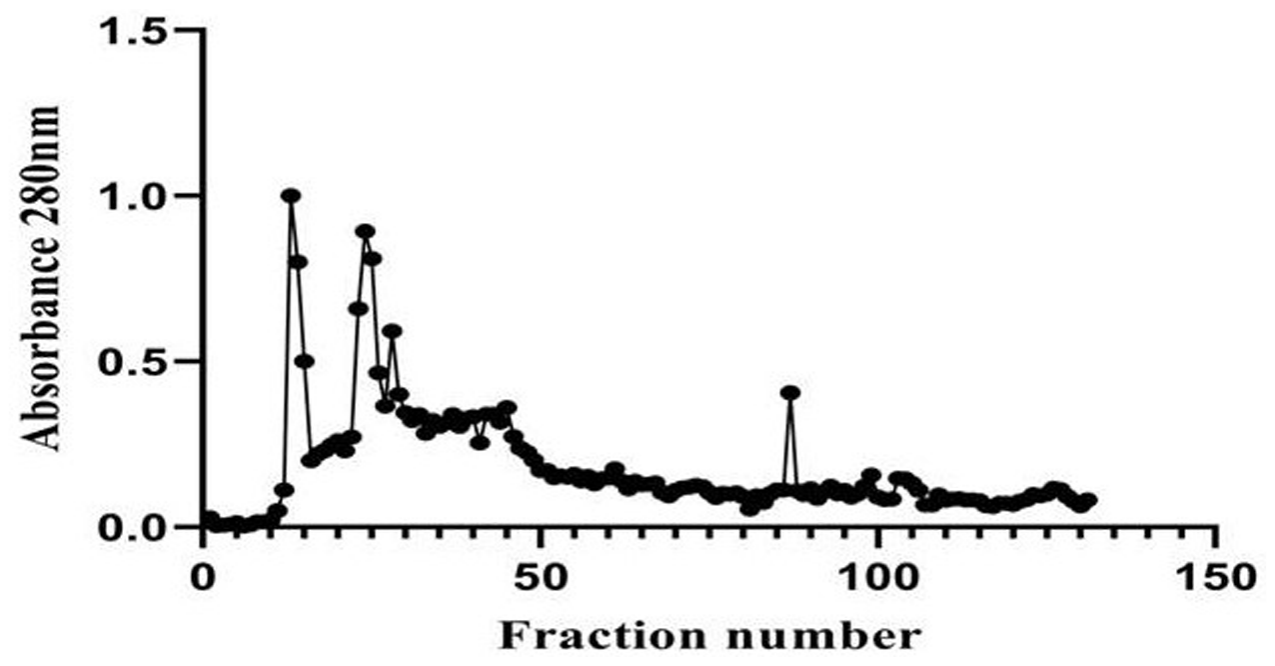
Elution profile of *Boudabousia marimammalium* culture protein extract using Sephadex G-10 column chromatography.

A 20 mg protein sample was loaded onto the column, and 2 mL fractions were collected, totaling ~216 mL (2.5 bed volumes). Absorbance at 280 nm was measured, and a graph was plotted with the fraction numbers (X-axis) and absorbance (Y-axis). The crude protein extract exhibited a concentration of 2.3 mg/mL, which was considered the baseline for purification (1-fold) with a yield of 100%. Following gel filtration chromatography using Sephadex G-10, the protein concentration was reduced to 0.1950 mg/mL. However, this step resulted in significant purification, achieving an 11.52-fold increase in purity, although with a reduced yield of 8.48% (Figure 5).

### Antimicrobial and anticancer activity

**Figure 6:**
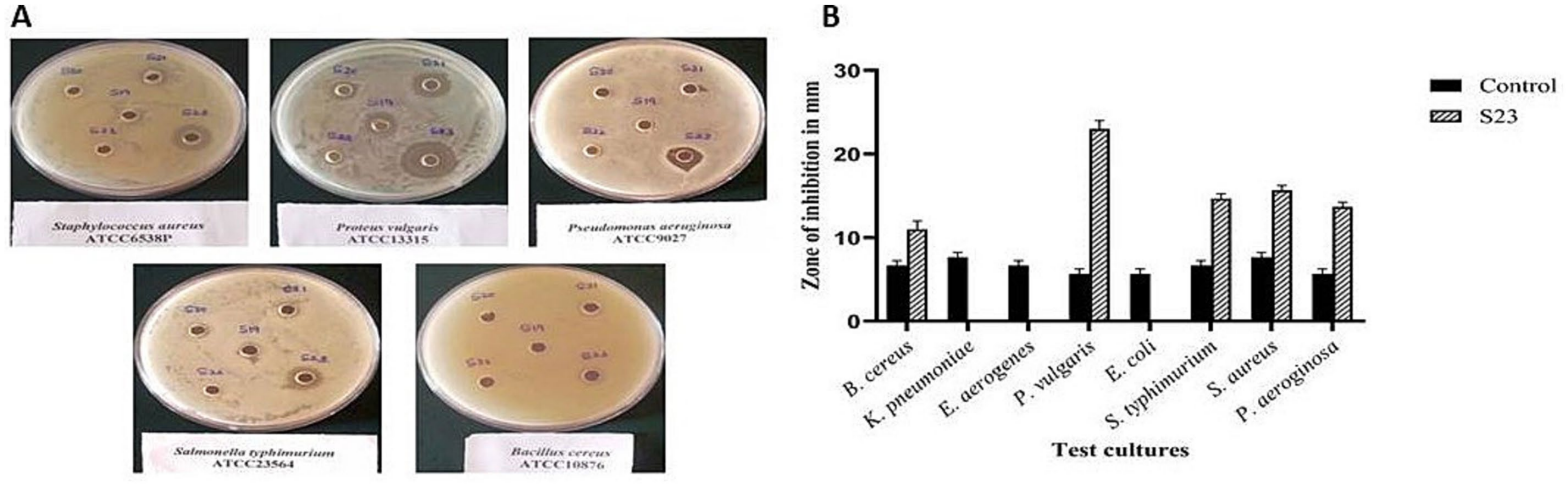
**(a)** Pictorial images of the antimicrobial activity of actinomycete isolate S23 against *Bacillus cereus, Proteus vulgaris, Salmonella typhimurium, Staphylococcus aureus,* and *Pseudomonas aeruginosa*, and **(b)** Graphical representation of the antimicrobial efficiency of *Actinomyces* isolate S23 on eight standard cultures.

The actinomycete isolate *Boudabousia marimammalium* showed effective antimicrobial activity, particularly against Gram-positive bacteria (*S. aureus* and *B. cereus*, Figure 6A) and Gram-negative bacteria (*P. vulgaris*, *S. typhimurium*, and *P. aeruginosa*). The bioactive peptides from this isolate outperformed S18-S22 isolates (Table 2) and demonstrated significant inhibition compared to standard antibiotics, such as streptomycin (Figure 6B).

**Table 2:** Antimicrobial potential (zone inhibition in mm) for partially purified protein extract of *Boudabousia marimammalium* culture.

Isolate	*B. cereus*	*K. pneumoniae*	*E. aeruginosa*	*P. vulgaris*	*E. coli*	*S. typhimurium*	*S. aureus*	*P. aeruginosa*
S18	--	--	--	--	--	--	--	--
S19	--	--	--	14 ± 0.6	13 ± 0.6	--	10 ± 0.6	--
S20	--	--	--	12 ± 0.6	14 ± 0.1	--	--	--
S21	10 ± 0.6	--	--	16 ± 1.0	--	--	10 ± 0.6	--
S22	--	--	--	8 ± 0.3	13 ± 0.3	--	--	--
S23	**10 ± 0.6**	**--**	**--**	**22 ± 1.0**	**--**	**14 ± 0.6**	**15 ± 0.6**	**13 ± 0.6**
Ampicillin	22 ± 0.5	20 ± 0.4	21 ± 0.6	23 ± 0.7	24 ± 0.3	21 ± 0.6	25 ± 0.4	19 ± 0.5

(−) indicates no inhibition observed; values are mean inhibition zones ± standard deviation (*n* = 3). Bold values depict the significant inhibition compound to standard antibiotics such as streptomyces.

**Figure 7:**
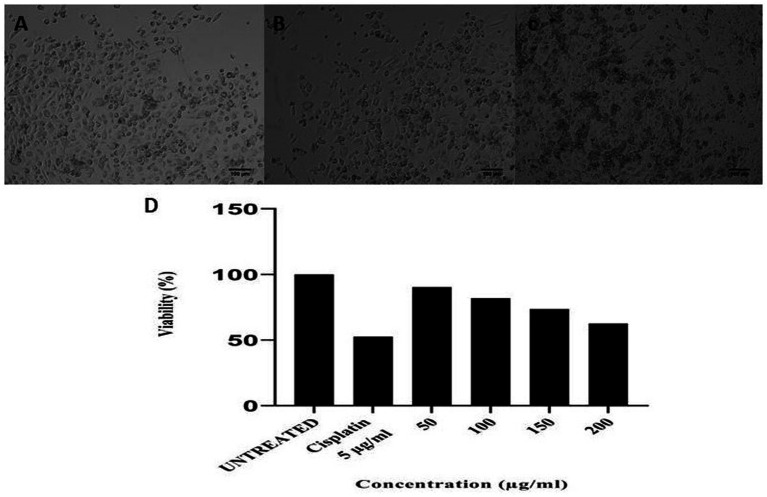
**(a)** Untreated PC3 cells, **(b)** 5 μg of cisplatin, **(c)** 200 μg of anticancer activity of *Boudabousia marimammalium* showed a potential of 37.43%, and **(d)** differential protein extraction efficiency of *Boudabousia marimammalium.*

Antimicrobial peptides (AMPs) from *B. marimammalium* also exhibited dose-dependent antiproliferative effects on PC3 prostate cancer cells, achieving 10–37% inhibition at 50–200 μg concentrations, compared to cisplatin (Figure 7). This dual antimicrobial and anticancer activity aligns with reported studies on actinomycete-derived peptides and supports their potential in therapeutic applications (Yin et al., 2008; Alapati and Muvva, 2013).

Antimicrobial peptides (AMPs) have been shown to exhibit cytotoxic effects against various cancer cell lines (Tornesello et al., 2020), including breast cancer, lung cancer, melanoma, leukemia, and lymphoma. These AMPs with anticancer activity are termed “anticancer peptides” (ACPs), and the shared structural and functional characteristics of AMPs and ACPs, such as cationicity and membrane-disrupting abilities, underlie their dual antimicrobial and anticancer activities (Guzmán-Rodríguez et al., 2015; Jafari et al., 2022; Qu et al., 2024).

### LC–MS analysis/peptide characterization

**Figure 8:**
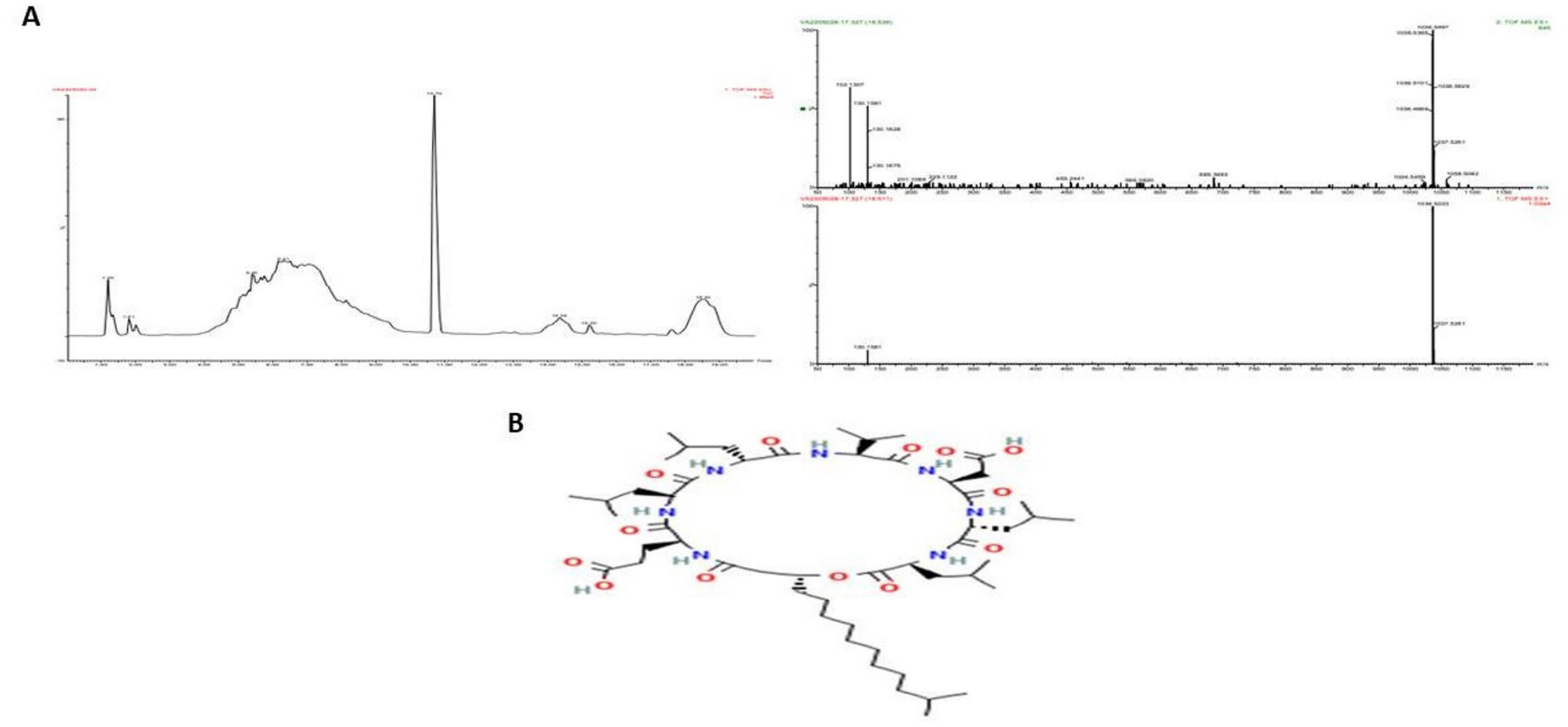
Elution profile of protein extracted from *Boudabousia marimammalium* YKIKM. MU03 protein. **(a)** Mass confirmation and analysis record, and **(b)** 2D structure of Tryprostatin B.

LC–MS analysis of the partially purified protein identified it as Tryprostatin B (Figure 8), a cyclic dipeptide with anticancer and antimitotic properties. Tryprostatin B, also known as deoxybrevianamide E, differs from Brevianamide F due to a prenyl group at the second position of the indole ring. It exhibits cytotoxic activity and plays a key role in the antimitotic pathway. Biochemical and genetic studies have identified biosynthetic genes, intermediates, and shunt products linked to its production.

## Discussion

The unique environmental conditions of the mangrove habitat in Kallapu, Mangalore, facilitated the isolation of *Boudabousia marimammalium*, a rare actinomycete species previously reported only in marine mammals in the UK (Hoyles et al., 2001). The soil’s physical characteristics and moderate pH (7.2) provided an ideal environment for actinomycete growth, as confirmed by the distinct morphological and pigmentation features of isolate S23. Growth optimization highlighted starch casein nitrate as the most suitable medium, yielding excellent growth and spore production. Similar findings have been reported, emphasizing the importance of tailored growth conditions for actinomycete cultivation (Subramani and Aalbersberg, 2012).

Molecular sequencing confirmed that S23 was *B. marimammalium* YKIKM. MU03 (GenBank accession number: MW898115). This is the first report of this species in mangroves, expanding its known habitat from marine mammals to terrestrial mangrove ecosystems. Phylogenetic analysis (Figure 4) and comparative species likelihood further validated this taxonomy. The rare occurrence of *B. marimammalium* highlights the potential of unexplored habitats, such as mangroves, for the discovery of novel actinomycete strains with therapeutic potential (Yu et al., 2024).

Although full-length 16S rRNA sequences (approximately 1,400–1,500 bp) are considered the gold standard for species-level identification, several studies have reported that partial sequences exceeding 900 bp can provide reliable genus- and sometimes species-level resolution, particularly when encompassing multiple hypervariable regions (Janda and Abbott, 2007; Chakravorty et al., 2007; Yarza et al., 2014). In our study, the 986 bp sequence covered key variable regions (V3–V9) and showed >99% identity to *Boudabousia marimammalium* in basic local alignment search tool analysis. Phylogenetic clustering of the type strains further supported this identification. Nonetheless, we acknowledge the limitations of using a partial sequence and recognize that near-full-length 16S rRNA sequencing provides additional taxonomic resolution. This issue will be addressed in future studies.

Protein extraction and purification processes demonstrated the isolate’s ability to produce bioactive compounds with significant antimicrobial and anticancer properties. Fraction 87, identified as the peak protein fraction with 8.48% yield and 11.52-fold purification, exhibited remarkable bioactivity. Actinomycete-derived peptides, including antimicrobial peptides (AMPs), have been widely reported to inhibit both Gram-positive and Gram-negative bacteria (Hamamoto et al., 2002). In this study, *B. marimammalium* showed potent activity against pathogens like *S. aureus, B. cereus, P. vulgaris, S. typhimurium,* and *P. aeruginosa* (Figure 6A). These pathogens are known to cause critical infections, including sepsis, typhoid fever, and respiratory diseases, underscoring the clinical relevance of these findings (Baron et al., 1992; Akova, 2016).

In addition to their antimicrobial effects, the bioactive peptides exhibited anticancer activity, as demonstrated by the dose-dependent inhibition of PC3 prostate cancer cells (Figure 7). At concentrations of 50–200 μg, the peptides achieved 10–37% antiproliferative effects, comparable to the standard drug cisplatin. These findings align with reports of actinomycete-derived anticancer peptides that exhibit dual antimicrobial and anticancer activities through membrane disruption and cytotoxic mechanisms (Zasloff, 2002; Papo and Shai, 2005; Hancock and Sahl, 2006).

LC–MS analysis identified the bioactive peptide as Tryprostatin B, a cyclic dipeptide with anticancer and antimitotic properties. Tryprostatin B is structurally similar to Brevianamide F but contains a prenyl group, enhancing its bioactivity. Previous studies have highlighted Tryprostatin B’s role in inhibiting mitotic pathways and its cytotoxic effects *in vitro* and *in vivo*. The biosynthetic pathways and genetic determinants associated with this peptide further underscore its therapeutic potential, as demonstrated by recent advances in microbial peptide research (Alkhalaf and Ryan, 2015; Wright, 2019; Yu et al., 2024).

This study provides compelling evidence for the antimicrobial and anticancer potential of *B. marimammalium* peptides, particularly Tryprostatin B, in a novel mangrove habitat. These findings contribute to the growing body of research on actinomycete-derived bioactive compounds and their applications in developing new therapeutic agents for infectious diseases and cancer.

Similar studies have been conducted on *Streptomyces* sp. MCCB267 is an endosymbiont in sponges. *Streptomyces* sp.*-*derived bioactive compounds showed promising anticancer activity against the lung cancer NCI-H460 cell line. The activity was characterized by nuclear apoptotic morphology, viz., shrinkage of cell nuclei, chromatin condensation, and nuclear fragmentation (Dhaneesha and Sajeevan, 2016).

In another study, *in vivo* testing of bioactive substances obtained from actinomycetes in an Egyptian environment led to a considerable increase in protection against harmful Acid-fast bacilli (AFB). Actinomycete-derived bioactive compounds can be used as food additives, to provide protection, and to cure endemic liver illnesses (El-Nekeety et al., 2017). Based on these findings, the actinomycete group may have some anticancer action against different cell types. Therefore, our study provides evidence that PC3 cells were successfully treated with peptides originating from the actinomycete group.

## Conclusion

Mangrove habitats, with their extreme and unique conditions, serve as hotspots for actinomycete diversification, fostering the production of bioactive compounds with therapeutic potential. Our study identified *Boudabousia marimammalium* as a rare actinomycete species, which was confirmed by molecular sequencing. This species exhibited remarkable features, including spherical spine spores and antimicrobial peptides (AMPs) with dual antimicrobial and anticancer activities. These AMPs, also termed “anticancer peptides” (ACPs), showed cytotoxic effects against the PC3 cell line and were partially purified as tryprostatin B, a known cyclic dipeptide with anticancer properties. The peptides’ functional characteristics, such as membrane disruption and cationicity, underlie their therapeutic efficacy. However, limited studies on *Boudabousia marimammalium* necessitate further research to explore its pharmacokinetics, pharmacodynamics, and full therapeutic potential.

## Data Availability

The original contributions presented in the study are included in the article/supplementary material, further inquiries can be directed to the corresponding authors.
